# Towards grapevine root architectural models to adapt viticulture to drought

**DOI:** 10.3389/fpls.2023.1162506

**Published:** 2023-03-14

**Authors:** Lukas Fichtl, Marco Hofmann, Katrin Kahlen, Kai P. Voss-Fels, Clément Saint Cast, Nathalie Ollat, Philippe Vivin, Simone Loose, Mariem Nsibi, Joachim Schmid, Timo Strack, Hans Reiner Schultz, Jason Smith, Matthias Friedel

**Affiliations:** ^1^ Department of General and Organic Viticulture, Hochschule Geisenheim University, Geisenheim, Germany; ^2^ Department of Modeling and Systems Analysis, Hochschule Geisenheim University, Geisenheim, Germany; ^3^ Department of Grapevine Breeding, Hochschule Geisenheim University, Geisenheim, Germany; ^4^ EGFV, University of Bordeaux, Bordeaux Sciences Agro, INRAE, ISVV, Villenave d’Ornon, France; ^5^ Department of Wine and Beverage Business, Hochschule Geisenheim University, Geisenheim, Germany; ^6^ Gulbali Institute for Agriculture, Water and Environment, Charles Sturt University, Orange, NSW, Australia

**Keywords:** vineyard, sustainability, root phenotypes, water, plant architecture, stress

## Abstract

To sustainably adapt viticultural production to drought, the planting of rootstock genotypes adapted to a changing climate is a promising means. Rootstocks contribute to the regulation of scion vigor and water consumption, modulate scion phenological development and determine resource availability by root system architecture development. There is, however, a lack of knowledge on spatio-temporal root system development of rootstock genotypes and its interactions with environment and management that prevents efficient knowledge transfer into practice. Hence, winegrowers take only limited advantage of the large variability of existing rootstock genotypes. Models of vineyard water balance combined with root architectural models, using both static and dynamic representations of the root system, seem promising tools to match rootstock genotypes to frequently occurring future drought stress scenarios and address scientific knowledge gaps. In this perspective, we discuss how current developments in vineyard water balance modeling may provide the background for a better understanding of the interplay of rootstock genotypes, environment and management. We argue that root architecture traits are key drivers of this interplay, but our knowledge on rootstock architectures in the field remains limited both qualitatively and quantitatively. We propose phenotyping methods to help close current knowledge gaps and discuss approaches to integrate phenotyping data into different models to advance our understanding of rootstock x environment x management interactions and predict rootstock genotype performance in a changing climate. This could also provide a valuable basis for optimizing breeding efforts to develop new grapevine rootstock cultivars with optimal trait configurations for future growing conditions.

## 1 Introduction

Grapevine (*Vitis vinifera* L.) is recognized as being well adapted to challenging environments (Ollat et al., 2019). With current climate change projections, however, abiotic stress in viticulture is likely to increase to levels that potentially jeopardize grape production, quality and wine typicity (Fraga et al., 2016; Schultz, 2016; Ollat et al., 2019). Temperatures and evaporative demand have risen and are expected to continue rising in many viticultural areas (Schultz, 2017). In addition, precipitation patterns are likely to be affected, with rainfall events becoming more erratic, resulting in increased frequency, severity and duration of drought periods and making water availability arguably one of the most crucial environmental factors limiting future growth and productivity of crops in general and viticulture in particular (Costa et al., 2016; Delrot et al., 2020; Gambetta et al., 2020; IPCC, 2021).

Winegrowers have high awareness of the effects of climate change. The vast majority of wineries in Europe (Loose and Pabst, 2019) and worldwide (Neethling et al., 2020) state that they have noticed climate change effects in the recent past, with a majority referring specifically to drought stress and water scarcity. Climate change effects are perceived as particularly detrimental in already hot and dry regions such as Spain and southern France (Neethling et al., 2020), and even in parts of the world with extensive irrigation infrastructure recent experience shows that water security is not guaranteed under extended drought (Van Dijk et al., 2013; Lund et al., 2018). In the case of Germany, growers perception of climate change effects on yield and quality has changed from positive (Battaglini et al., 2009) to overwhelmingly negative in the past decade (Loose and Kiefer, 2020), with drought risk in steep slope viticulture (Strub and Loose, 2021) and young vineyards perceived as particularly critical scenarios (Friedel et al., 2022).

There is a wide diversity of adaptation levers to improve the management of viticulture under future climatic conditions (Naulleau et al., 2021). Among them, the choice of existing or breeding of novel rootstocks suitable for site-specific environmental characteristics represents an elegant way ensuring adaptation to a range of abiotic and biotic stresses (Delrot et al., 2020), while maintaining traditional scion varieties that are familiar to the market (Ollat et al., 2016; Zhang et al., 2016). There is wide consensus that rootstocks will be central to adapting to the challenges of climate change, notably the rising risk of drought stress, with both choice of the right plant material and breeding of new rootstock genotypes presenting key strategies (Marguerit et al., 2012; Corso and Bonghi, 2014; Berdeja et al., 2015; Grossi et al., 2016; Ollat et al., 2016; Delrot et al., 2020; Gambetta et al., 2020). The great diversity of existing rootstocks, however, still remains widely underexploited in viticultural practice (Ollat et al., 2016), possibly due to a lack of decision support for growers regarding the choice of rootstocks (e.g. for Germany: Friedel et al., 2022). The choice of adequate rootstock varieties to reduce drought stress is a challenging task, as plant performance under drought is subject to strong genotype x environment (G x E) interactions (Tardieu, 2012). Grapevine drought tolerance is a particularly complex integrative trait with multiple underlying physiological mechanisms subject to rootstock x scion x environment x management (G_1_ x G_2_ x E x M) interactions. Such complexity and lack of mechanistic understanding of many drought responses can also prevent accurate prediction of drought stress risk under future climatic scenarios (Gambetta et al., 2020).

There is a general consensus in the plant research community that, among plant traits that play a role in drought stress physiology, root system architecture stands out as being of utmost relevance (Wasson et al., 2012; Comas et al., 2013; White et al., 2013; Lynch, 2018; Shoaib et al., 2022). The importance of root system architecture (i.e. the spatial distribution and shape of different root types within a volume of soil) and its temporal development lies in the fact that water is heterogeneously distributed in the soil in space and time. The spatio-temporal deployment of roots will therefore substantially determine the ability of plants to take up water (De Dorlodot et al., 2007; Rogers and Benfey, 2015; Tron et al., 2015). The challenge is that root architecture traits are complicated to assess in a meaningful spatial and temporal resolution, particularly in perennials grown under field conditions (Dumont et al., 2016).

To better match rootstocks to target growing areas, it is necessary to combine detailed knowledge of current and future drought stress scenarios with an understanding of root architecture traits that may contribute to drought tolerance (White et al., 2013). In that sense, modeling can assist with explaining observed data, testing hypotheses and integrating drought conditions and plant performance on the scale of individual plants up to crop stands and deepen our understanding of the complex high-dimensional space of G x E x M interactions (Soualiou et al., 2021). Root architecture models can assist in our understanding how roots access and extract soil resources. They enable researchers to plan and interpret the results of root sampling strategies and help to explore how single or sets of root architecture traits contribute to drought adaptation within various growing scenarios, without the necessity of executing numerous experiments that would be required to display the vast array of soil and drought conditions found in agricultural regions (Dupuy et al., 2010; Schnepf et al., 2018a; Schnepf et al., 2018b). This can also provide a basis to formulate breeding targets for the development of improved rootstock cultivars with desired trait configurations (Cooper et al., 2021).

In this perspective, we review state-of-the-art knowledge from different disciplines and propose an approach to bridge knowledge gaps in the role of grapevine root system architecture under drought, with an emphasis on dynamic root development of young vines. We investigate how a highly interdisciplinary collaboration with a strong focus on modeling might enhance our understanding of spatio-temporal interplays of both soil water availability and root architecture, and thereby identify modeling strategies that may advance our understanding of grapevine rootstock traits. Such an integrative approach would facilitate knowledge transfer into viticultural practice and broaden the possibilities of decision support for winegrowers. It could also help to improve rootstock genetic improvement programs that target specific future environmental scenarios.

## 2 Simulating realistic environments for rootstocks

Environmental effects and G x E interactions have been shown to be larger sources of variance for target traits than genotype effects alone, particularly under stress conditions (Chenu, 2015). Hence, detailed knowledge on the spectrum of drought stress scenarios representative for the majority of future vineyard situations is necessary to advise growers on their choice of rootstock and inform breeders about the requirements rootstocks will have to meet in the course of climate change. Such drought stress scenarios can be adequately characterized for different environments by modeling the vineyard water balance (Hofmann et al., 2022).

The capacity to model the vineyard water balance based on observed or simulated weather data (e.g. Lebon et al., 2003; Pieri et al., 2012; Gaudin et al., 2014; Hofmann et al., 2014) is also of high importance for the identification of traits with high agronomic relevance, and to define trait combinations in new rootstocks with improved performance in future vineyards. For example, Hofmann et al. (2022) modeled future drought stress risk on a vineyard plot scale for two winegrowing regions using an ensemble of climate change projections. In their model, topographical, geological, meteorological and vineyard management factors (e.g. slope and aspect, cover crop use, row spacing) were integrated to obtain precise predictions of the vineyard water balance. While such model approaches fulfill the requirements for the description of future drought stress scenarios for established vineyards, they do not specifically use root parameters such as rooting depth or root length density (RLD), and assume that roots can extract water from the complete soil reservoir defined by a default effective root zone. Further, soil water content is modeled only by the fraction of the available water capacity without considering the vertical distribution of soil water. Hence, their use for specific predictions such as young vine survival might be limited, as only a fraction of soil water may be available for young vines due to limited rooting depth and radius. Under the assumption that water extraction of young vines can be represented by limiting the effective root zone, the model was applied to predict the water balance of young vineyards, but such predictions remain to be validated by targeted experiments (Hofmann et al., 2022). As genetic variability for root parameters has been shown to exist (e.g. Tandonnet et al., 2018), extended models that are capable of capturing a range of root trait configurations would provide the opportunity to consider root genetic diversity in the modelling process.

**Figure 1:**
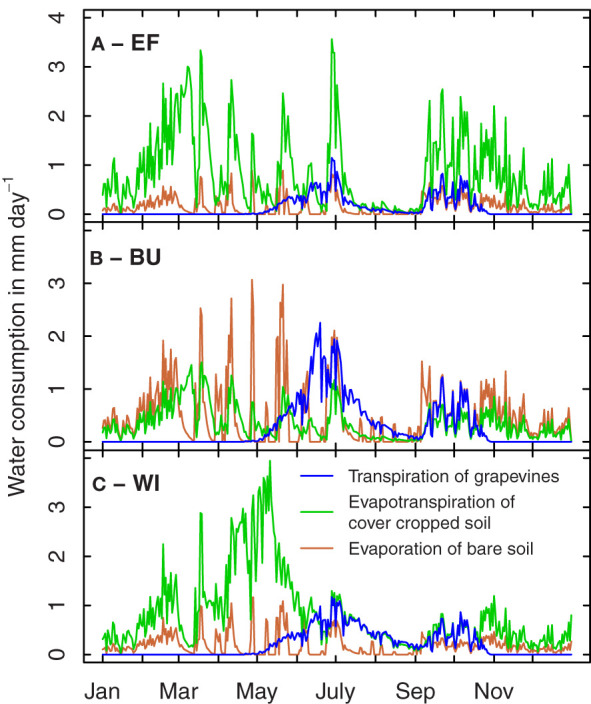
Example simulation of grapevine transpiration, evapotranspiration of cover cropped soil and evaporation of bare soil for three vineyards in the Rheingau region in 2022 **(A–C)**. **(A)** Ehrenfels vineyard, row spacing 2.50 m, 85 mm available water capacity (AWC), fully cover cropped with exception of the undervine area (width 0.4 m). **(B)** Burgweg vineyard, row spacing 1.60 m, 115 mm AWC, every other row cover cropped. **(C)** Wilgert vineyard, row spacing 1.60 m, 320 mm AWC, fully cover cropped with exception of the undervine area. Development of cover crops is divided into several growth stages.

Due to the high variability of topologic and geologic parameters typically associated with many traditional winegrowing regions, and a high variability of monetary and cultural value of vineyards often located in close spatial proximity, a plot-scale resolution as chosen by Hofmann et al. (2022) seems adequate for water balance simulations in viticulture. Water balance modeling has also shown the importance of E x M interactions for the water balance of target growing environments and demonstrated the particular importance of cover cropping (Celette et al., 2010; Gaudin et al., 2014; Hofmann et al., 2014). **Figure 1** illustrates simulated annual courses of water consumption by grapevine transpiration, evapotranspiration of cover cropped soil and evaporation of bare soil of three different vineyards located in close spatial proximity. In the extreme example illustrated in **Figure 1A** (steep slope, southern inclination, shallow soil, wide, cover-cropped rows), a large fraction of the available water is transpired by the cover crop before the grapevines start to consume water. This could lead to early drought stress during shoot development and flowering in dry springs. As cover cropping practice differs substantially among dry farmed winegrowing regions (e.g. Celette et al., 2008; Abad et al., 2021), the inclusion of a cover crop component in modeling drought stress scenarios is fundamental to apply water balance models on a large scale and may justify further model refinements such as the type of cover crop used.

In perspective, relevant drought stress scenarios might be extracted from a cluster analysis (e.g. Mishra and Singh, 2011; Crespo-Herrera et al., 2021) performed on the results of a large scale application of water balance models combined with ensembles of climate change projections (Chenu, 2015). Input data needed to run water balance models on large scales with high (plot-scale) spatial resolution have become increasingly available in the past decades by use of digital elevation models and the increased availability of high-resolution soil maps for many European regions (Panagos et al., 2022). Data on soil water availability may also become available in higher spatial (i.e. horizontal and vertical) resolution using novel techniques such as cosmic ray neutron sensing (Baroni et al., 2018).

## 3 Root architecture is a key determinant of grapevine performance under drought

Grapevines can cope with water deficits through a range of mechanisms that help to delay the onset of severe water stress, and through mechanisms that help the plant tolerate more negative water potentials without significant tissue damage (Chaves et al., 2010; Tsegay et al., 2014; Lovisolo et al., 2016; Simonneau et al., 2017; Gambetta et al., 2020). In this regard, various single traits have been discussed to play a pivotal role in grapevine drought adaptation, including an array of morphological, anatomical and physiological characteristics of both aerial and underground organs (Simonneau et al., 2017; Gambetta et al., 2020). Although difficult to rank according to their importance for rootstock drought tolerance with the current state of knowledge, a large body of evidence suggests that root architectural traits, the temporal pattern of their deployment and their plasticity in response to soil water availability seem crucial parameters to estimate rootstock performance in any drought prone area (e.g. Tsegay et al., 2014; Ollat et al., 2017; Tandonnet et al., 2018). Despite the extensive knowledge gained in these studies, our knowledge on rootstock architecture in the field remains limited due to the difficulty in accessing the root system of the vine, which restricts phenotyping throughput (Soar and Loveys, 2007; De Herralde et al., 2010; Tandonnet et al., 2010; Dumont et al., 2016; Archer and Saayman, 2018; Tandonnet et al., 2018; Ollat et al., 2019). Knowledge about early root development and root morphology of grapevines grown in the field and particularly their relationship with vine performance in established vineyards, root growth plasticity and root growth dynamics seems particularly limited (Ollat et al., 2017). This prevents a better understanding of root deployment in the field, an important step to address issues with practical relevance such as grower uncertainties regarding the optimal rootstock choice to support the survival of young vines.

A range of traits are commonly used in the literature to describe grapevine root architecture and growth (**Table 1**), among them static root traits (i.e. measurable at a single point of time) and dynamic root traits (i.e. related to spatio-temporal changes, De Dorlodot et al., 2007).

**Table 1:** Overview of root architecture traits commonly used in grapevine research.

Trait	Unit	References
rooting angle	°	Smart et al., 2006; De Herralde et al., 2010; Fort et al., 2017; Cochetel et al., 2019; Schmitz et al., 2021
rooting depth	cm; m	Morlat and Jacquet, 2003; Smart et al., 2006; De Herralde et al., 2010; Tsegay et al., 2014; Hunter et al., 2016; Kocsis et al., 2016; Fort et al., 2017; Cochetel et al., 2019
total root length	mm; cm; m	Bassoi et al., 2003; De Herralde et al., 2010; Alsina et al., 2011; Dumont et al., 2016; Kocsis et al., 2016; Ollat et al., 2017; Peccoux et al., 2018; Yıldırım et al., 2018; Peiró et al., 2020; Schmitz et al., 2021; Burgess, 2022
root length density	mm/cm^3^; cm/cm^3^; m/m^3^	Bassoi et al., 2003; Soar and Loveys, 2007; Tsegay et al., 2014; Peccoux et al., 2018; Burgess, 2022
root length area	cm/cm^2^	Peccoux et al., 2018
root density	no./m^2^	Morlat and Jacquet, 2003; Hunter et al., 2016; Ferlito et al., 2020
root diameter	mm; cm	Bauerle et al., 2008; De Herralde et al., 2010; Tsegay et al., 2014; Barrios-Masias et al., 2015; Dumont et al., 2016; Kocsis et al., 2016; Peccoux et al., 2018; Peiró et al., 2020
total number of roots	no.	Morlat and Jacquet, 2003; Bauerle et al., 2008; Comas et al., 2010; Hunter et al., 2016; Kocsis et al., 2016; Tandonnet et al., 2018; Cochetel et al., 2019; Schmitz et al., 2021
root biomass	g	Bassoi et al., 2003; Morlat and Jacquet, 2003; De Herralde et al., 2006; Soar and Loveys, 2007; De Herralde et al., 2010; Tandonnet et al., 2010; Gambetta et al., 2012; Dumont et al., 2016; Hunter et al., 2016; Fort et al., 2017; Tandonnet et al., 2018; Yıldırım et al., 2018; Ferlito et al., 2020; Bartlett et al., 2021
root volume	m^3^	Soar and Loveys, 2007; Jones, 2012; Gambetta et al., 2020; Peiró et al., 2020; Schmitz et al., 2021
root surface area	cm^2^; m^2^	Bassoi et al., 2003; Gambetta et al., 2012; Dumont et al., 2016; Yıldırım et al., 2018; Peiró et al., 2020; Bartlett et al., 2021; Cuneo et al., 2021
specific root length	m/g	Pérez-Harguindeguy et al., 2013; Zhu et al., 2021
rooting index	no. of roots < 2 mm/no. of roots > 2 mm	Swanepoel and Southey, 1989; Ferlito et al., 2020
ramification/number of lateral roots/branching frequency	no.; no./volume; no./branching point	De Herralde et al., 2010; Cochetel et al., 2019; Schmitz et al., 2021
root growth plasticity	mm/cm^2^ per season	Bauerle et al., 2008
root growth/elongation rate	mm/d; cm/d; mm/h	Dumont et al., 2016; Mahmud et al., 2018; Cuneo et al., 2021
root production pattern	no./period; mm/period; cm/period	Comas et al., 2005; Bauerle et al., 2008; Comas et al., 2010; Fort et al., 2017

Many of these root traits play a context-dependent role in the drought tolerance of grapevines. For instance, in intermittent drought scenarios prevailing in most central European winegrowing regions, the ability to take up water in topsoil when summer precipitation becomes available (either by maintaining or reinitiating growth of fine roots and high total root length density) is likely to contribute to drought tolerance (Cuneo et al., 2021), but such traits would be of limited value in storage-driven hydrology typical of vineyards in Mediterranean climates, in which rooting depth seems to play a crucial role (Tron et al., 2015).

Soil water depth is another major parameter of the drought stress scenario and determines the strategy to ensure plant performance. In soils with available deep water, a strategy to outgrow the water deficit might be best suited for plant survival and productivity (e.g. high root length density at depth). In rather shallow soils or soils where no deep water is available, a reduction of metabolic investment into root development may be beneficial for vine survival, since a trade-off exists between the carbon costs of root systems and the benefit of increased water uptake under drought, limiting the necessity of investing into large root system under specific growing conditions (Tardieu et al., 2017). It is, however, unclear whether such parsimonious strategies will benefit vine survival and productivity in case there is cover crop competition (see **Figure 1**).

Management practices such as planting density, cover cropping strategy or canopy management, and soil parameters such as penetration resistance also exert a strong role in shaping root system architecture and its development (Richards, 1983; Reimers et al., 1994; Smart et al., 2006; Celette et al., 2008; Hunter et al., 2016). Describing the complex interplay of rootstock genotypes and their interactions with various environment and management factors would require an enormous amount of field phenotyping studies - an impossible task considering the difficulties in accessing the root network.

To get a better understanding of the complex interactions shaping root growth, a combination of phenotyping methods with increased throughput or increased spatio-temporal resolution and advanced modeling is suggested in the following paragraphs.

## 4 Phenotyping techniques to capture root system development

Root phenotyping studies in the field and, under significant limitations, in the greenhouse, are needed to capture root architecture traits under conditions as close as possible to practical viticulture. In the following section, we briefly discuss methods to capture root architectural traits and discuss their advantages and drawbacks.

Rhizoboxes and rhizotrons (hereinafter referred to as rhizoboxes) are specialized growth chamber systems. Usually, simple designs comprising a frame, at least one transparent pane and an opaque cover are used to monitor the temporal below-ground development of young grapevine plants, cuttings or seedlings grown in a soil-like medium in a non-destructive way and to characterize a range of static and dynamic root architecture traits in a limited space, yet basically in 2 dimensions (Dumont et al., 2016; Baldi et al., 2018; Krzyzaniak et al., 2021; Yee et al., 2021). The benefit of this simple and cost-effective approach is the investigation of root growth under controlled conditions with low space requirement and high throughput. However, there are limitations in the use of rhizoboxes in perennial plants like grapevines. In particular, the design of rhizoboxes restricts root growth (e.g. maximum rooting depth, 2D), thus determining the boundaries of the experiment spatially and temporally (Poorter et al., 2012).

To phenotype root systems of field grown grapevines with minimal soil disturbance, soil coring and minirhizotrons provide complementary methods for characterizing spatio-temporal differences in root growth traits (e.g. Soar and Loveys, 2007; Linsenmeier et al., 2010). These techniques are particularly suited for studies where comparisons of multiple genotypes or locations are of interest, or where there is a requirement to follow the development of root systems with repeated observations at a limited spatial resolution (Soar and Loveys, 2007; Bauerle et al., 2008). The information obtained from soil coring should be complemented with soil moisture monitoring in assessing the functional implications of measured root distribution and can assist in parametrizing water balance models where depth of water uptake and relative share of soil water with cover crops can be used as inputs (Hofmann et al., 2014; Zhu et al., 2021). For minirhizotrons, the ability to make more frequent observations down to a scale of individual roots provides further functional insight by allowing detailed assessments of root elongation rates, root lifespan, and seasonal fine root growth dynamics (e.g. Comas et al., 2010; Savi et al., 2018). Aspects of both methods are labor intensive, but technological developments in image collection and analysis, as well as opportunities to apply molecular techniques in the study of soil and roots collected from cores is greatly increasing the value of information that can be obtained (Haling et al., 2011; Lobet et al., 2013). For example, molecular techniques could allow to reliably discriminate cover crop from grapevine roots and hence obtain information about the spatial distribution of the roots of multiple species in a cover cropped vineyard. In addition, inverse estimation methods have been used to derive root architecture traits such as maximum length, elongation rate, insertion angles, and numbers of zero-order roots from soil coring (Morandage et al., 2021), a strategy also applicable to minirhizotron data (Schnepf et al., 2018a).

Root architecture traits of field grown grapevines can be acquired at very high spatial resolution by excavating entire root systems followed by 3D-digitization (e.g. using a low magnetic field digitizer such as Fastrak, Polhemus, Colchester, VT, USA). 3D-digitization has successfully been applied to phenotype aboveground grapevine growth (Schmidt et al., 2019) and to digitize root architecture of different tree species (Danjon et al., 2005; Danjon et al., 2013; Danquechin Dorval et al., 2016), but to the best of our knowledge has not been applied to root systems of grapevines. Manual excavation is laborious, whereas removing the soil with high-pressure air is efficient without harming fine and coarse roots (Danjon et al., 2005). Manual uprooting can be done for smaller plants, but for larger plants, mechanical uprooting using a mechanical shovel is generally much more rapid. In this case, the number of roots lost during uprooting is large in the peripheral part of the root system. Once excavated, the root system can be measured *in situ* or brought to the laboratory, provided that the roots are rigid enough to maintain the overall 3D structure of the root system. *In situ* measurement is specifically suitable for young plants to reduce the loss of roots and to accurately measure root system geometry. After the excavation or uprooting the root system can be digitized. According to the downstream data distribution method, the digitized root geometry data are available in different formats: simple lists, structured lists (compare Schmidt et al., 2019) or multiscale tree graph (MTG) format file (Godin and Caraglio, 1998), where the root system is defined as a set of root axes subdivided into segments. Although both parts of this method, excavation and digitization, are very time-consuming and come at the expense of throughput, the high-resolution data output along with a functional annotation makes such data sets ideally suited for the integration into root growth models.

Of the methods described here, only rhizoboxes allow for a throughput in the scale needed to run genetic studies on root traits and/or screen larger breeding collections. Connecting field-based observations taken from older vines to seedling and/or cutting-based root measurements (e.g. on adventitious roots), for example *via* genetic correlation analyses, would provide highly valuable information for breeding regarding potential proxy-traits that genetic improvement programs could target at much higher throughput.

## 5 Modeling root growth

Root phenotyping data may be used to inform or parametrize models that might advance our understanding of the interaction of root architecture and drought stress response in specific environments, both for young and mature grapevines. This can be achieved by integrating traits of individual rootstocks into existing models, by the extension of existing models and by the parameterization of new root growth models. Root growth models describe growth of roots over time – often in relation to drivers such as water availability or nutrients. In this respect, such models are often part of classical crop models (e.g. APSIM, Keating et al., 2003). They typically consider root growth processes in relation to soil depth, focusing on one dimension only. Models for root architecture explicitly consider positioning of root segments in the soil, either in 2D or in 3D (e.g. Leitner et al., 2010; Postma et al., 2017; Barczi et al., 2018; Schnepf et al., 2018a; Schnepf et al., 2018b; Morandage et al., 2021). A root architectural model might belong to the class of functional-structural model (FSPM), if it integrates interactions with physiological processes. Functional-structural models may be static or dynamic over time. A dynamic FSPM includes both the growth and development (appearance) of new organs (Buck-Sorlin, 2013).

Among existing plant growth models that include root architectural traits, SurEau (Cochard et al., 2021) and APSIM grapevine (Zhu et al., 2021) are examples for a mechanistic plant model and a crop model, respectively, that allow the integration of a variety of rootstock traits. SurEau was developed to test the effect of drought stress on woody species hydraulics in the soil-plant-atmosphere continuum, and has been applied to study the effects of drought stress on hydraulic failure of several grapevine scion genotypes (Dayer et al., 2020; Dayer et al., 2022). Soil in SurEau is divided into several layers, each with its own root distribution. It however seems to have limitations in the application to vineyard situations, as it does not consider the row structure of the vineyard, or effects of cover cropping. APSIM is a modeling framework that has recently been parametrized for grapevine (Zhu et al., 2021). It can integrate several root traits (e.g. rooting depth; biomass accumulation; root length density; fine root distribution in specific vineyard zones like the inter-row space) in specific soil layers. One limitation of APSIM grapevine is that the variety of drought stress functions available in the parent framework have not been integrated into APSIM grapevine yet.

Further progress can be achieved by extending or modifying existing models. Modifying models such as SurEau to represent vineyard situations more accurately (e.g. by introducing row structure or a cover crop module), and possibly integrate a larger number of root traits will greatly expand our possibility to analyze drought damage to grapevines as a function of rootstock genotype. Similar output, but with a stronger focus on vineyard water balance on a large scale, might be obtained by expanding existing water balance models such as the one published by Hofmann et al. (2022) with root architecture traits. If an interface between physiological models (e.g. SurEau) and vineyard water balance models can be achieved, it may become possible to simulate vine hydraulic failure risk on a regional scale as a function of scion and rootstock, provided that scion/rootstock interactions are known.

The application of such models, however, would have the drawback that they do not yet integrate dynamic root traits nor root system architectural traits *sensu stricto*. Frameworks such as APSIM contain features that may allow for a dynamic simulation of root growth. Additional knowledge can be gained from the application of dynamic FSPMs, which are able to represent the development of plant architecture in time and space. Generic root FSPMs such as CRootBox (Schnepf et al., 2018a; Schnepf et al., 2018b; Zhou et al., 2020; Morandage et al., 2021), OpenSimRoot (Postma et al., 2017) or DigR (Barczi et al., 2018) may be used to predict the architecture of mature vines from young vine phenotyping data and are thus ideally suited to transfer results from limited greenhouse studies (e.g. in rhizoboxes) to the field. Also, FSPMs have already successfully been applied to model intercropping systems (Bourke et al., 2021 and references therein) and will hence be ideally suited to simulate root development of grapevines and cover crops, as well as their mutual interaction. For the parametrization of FSPMs, data obtained by 3D-digitisation are optimally suited (Schmidt et al., 2019; Schmidt et al., 2022). The application of FSPMs would additionally require a rather detailed representation of the spatial heterogeneity of soil water content due to its influence on the direction of root growth (De Dorlodot et al., 2007). The environments for *in silico* studies with grapevine FSPMs can be generated by water balance models with high spatial resolution. Ideally, such models could integrate a variety of drought tolerance related traits of below- and aboveground parts, but such models will be extremely complex and computationally expensive. To simplify the complex interplay of soil water balance and root architecture, Tron et al. (2015) linked a 1D-water balance model to a 3D-dynamic root growth model (Leitner et al., 2010) by downscaling 3D root data to a 1D sink term.

The high requirements on phenotyping and soil data may explain that FSPMs have not yet been used to simulate grapevine root growth so far. However, first above-ground FSPMs for grapevine already exist (e.g. Zhu et al., 2018; Schmidt et al., 2019) and are successfully applied to predict growth and plant water status under varying environments, demonstrating the potential of FSPMs as a powerful tool for grapevine rootstock research in the future.

## 6 Rootstock architectural models to guide predictive breeding

Given the critical role of rootstocks for grapevine performance under abiotic and biotic stresses, rootstock breeding is gaining an increasing attention as a strategy to tackle the impacts of climate change. Extending rootstock breeding pipelines to incorporate physiological modelling could enable a more informed definition of future breeding targets with the goal to deliver performance improvement under forecasted climatic fluctuations. Developing high-throughput phenotyping tools that enable population screenings for key root traits used as model parameters will be critical in order to integrate both physiological and genetic modelling in future rootstock genetic improvement programs. Modern breeding tools such as genomic selection that uses dense genomic marker maps (Meuwissen et al., 2001), or phenomic selection that uses non-destructive high-throughput phenotyping data (e.g. from hyperspectral imaging, Rincent et al., 2018) to predict the genotypic value of individuals for traits of interest are particularly promising. With the broad range of modelling approaches available, genomic and phenomic selection could be directly coupled with spatio-temporal modelling of physiological processes in order to better capture impacts of G x E interaction on crop performance (e.g. Cooper et al., 2014; Technow et al., 2015). Such transdisciplinary approaches would enable a more targeted exploration of the highly complex multi-dimensional G x E x M space with the potential to identify workable breeding paths that deliver novel solutions for performance improvement under climate change (Cooper et al., 2021).

## 7 Conclusion

The choice of drought tolerant grapevine rootstocks presents a viable means of adapting viticulture to relevant drought scenarios prevailing in winegrowing regions. The current state of knowledge allows us to simulate future drought stress scenarios of vineyards with a high spatial resolution and to predict scion transpiration and mortality risk as a function of water uptake by the roots. The knowledge of rootstock traits, however, still impedes predicting the role of rootstock genotypes in grapevine drought tolerance under given growing conditions (i.e. drought scenario, soil properties, management decisions). This gap of knowledge has so far hindered the knowledge transfer into practical viticulture and rootstock breeding, potentially explaining why the majority of the existing variety of rootstocks are only scarcely used in practice.

Although our knowledge on the importance of individual or sets of traits relevant for the drought tolerance of a grapevine rootstock (or conferred by it) is far from comprehensive, a large body of evidence points towards the high importance of root architectural traits, such as rooting depth, root length density or specific root length. Data on the spatio-temporal development of root architecture are still extremely scarce, considering the large variability of root growth brought about by differences in soil structure, and hence the need for a relatively large database to provide robust information. To increase available knowledge on root structure and development, as well as to characterize root growth modification by grapevine x cover crop interactions, rhizoboxes, minirhizotrons, soil coring and excavation/digitization are methods that have the potential to increase throughput or spatial/temporal resolution. Data on the spatio-temporal pattern of grapevine root development can be used as model inputs to evaluate the effects of root architectural traits on resource acquisition during root development in a given drought stress scenario. FSPMs seem ideally suited for this task. An integration of additional drought related traits as well as aboveground plant growth and function into crop or plant models may in the future provide for a comprehensive understanding of drought related traits for rootstock and overall grapevine performance and survival under water deficit. While such knowledge would represent a milestone in grapevine drought stress physiology, it would still need to be integrated into a highly interdisciplinary network of experts involving agronomists, soil scientists, climatologists, modelers, plant physiologists and plant geneticists that provides decision support for the sustainable climate change adaptation of viticulture.

## Data availability statement

The original contributions presented in the study are included in the article/supplementary material. Further inquiries can be directed to the corresponding author.

## Author contributions

LF and MF designed the overall concept of the manuscript. All authors contributed to the article and approved the submitted version.
